# Effects of estrogen on functional and neurological recovery after spinal cord injury: An experimental study with rats

**DOI:** 10.6061/clinics/2015(10)08

**Published:** 2015-10

**Authors:** Olavo Biraghi Letaif, Alexandre Fogaça Cristante, Tarcísio Eloy Pessoa de Barros Filho, Ricardo Ferreira, Gustavo Bispo dos Santos, Ivan Dias da Rocha, Raphael Martus Marcon

**Affiliations:** I Hospital das Clínicas da Faculdade de Medicina da Universidade de São Paulo Instituto de Ortopedia e Traumatologia, (IOT-HCFMUSP), Divisão de Cirurgia de Coluna Vertebral, Laboratório de Investigação Médica, São Paulo/SP, Brazil; II Hospital das Clínicas da Faculdade de Medicina da Universidade de São Paulo, Instituto de Ortopedia e Traumatologia, (IOT-HCFMUSP), Laboratório de Investigação Médica (LIM-41), São Paulo, SP, Brazil

**Keywords:** Spinal Cord Injuries, Estrogen, Central Nervous System/Injuries, Rats

## Abstract

**OBJECTIVES:**

To evaluate the functional and histological effects of estrogen as a neuroprotective agent after a standard experimentally induced spinal cord lesion.

**METHODS:**

In this experimental study, 20 male Wistar rats were divided into two groups: one group with rats undergoing spinal cord injury (SCI) at T10 and receiving estrogen therapy with 17-beta estradiol (4mg/kg) immediately following the injury and after the placement of skin sutures and a control group with rats only subjected to SCI. A moderate standard experimentally induced SCI was produced using a computerized device that dropped a weight on the rat's spine from a height of 12.5 mm. Functional recovery was verified with the Basso, Beattie and Bresnahan scale on the 2^nd^, 7^th^, 14^th^, 21^st^, 28^th^, 35^th^ and 42^nd^ days after injury and by quantifying the motor-evoked potential on the 42^nd^ day after injury. Histopathological evaluation of the SCI area was performed after euthanasia on the 42^nd^ day.

**RESULTS:**

The experimental group showed a significantly greater functional improvement from the 28^th^ to the 42^nd^ day of observation compared to the control group. The experimental group showed statistically significant improvements in the motor-evoked potential compared with the control group. The results of pathological histomorphometry evaluations showed a better neurological recovery in the experimental group, with respect to the proportion and diameter of the quantified nerve fibers.

**CONCLUSIONS:**

Estrogen administration provided benefits in neurological and functional motor recovery in rats with SCI beginning at the 28^th^ day after injury.

## INTRODUCTION

The treatment of spinal cord injuries (SCI) remains challenging; none of the available treatments are considered particularly effective 1 and SCI is still considered an irreversible condition 2. Although surgical mechanical stabilization is performed in unstable spinal fractures (with poor results), pharmacological treatments represent the most well-studied experimental protocol 3,4. Many pharmacological agents have been studied 2, and estrogen has shown consistent results as a neuroprotective agent 7-10.

Estrogen inhibits inflammation and activates a variety of cysteine proteases in animal models. Furthermore, estrogen shows anti-inflammatory activity in the cascade of events after SCI, including microglial activation, increased blood flow to the injured tissue, increased anti-apoptotic protein levels and attenuated post-traumatic influx of calcium, thus acting as a neuroprotective agent 7,.

More studies are necessary, however, to elucidate the role of estrogen in the reduction of secondary lesions after spinal cord trauma. Even if the spinal cord does not completely recover, the patient's quality of life can be improved through smaller tissue repairs that allow minimal functional recovery. Even if the patient cannot walk, their ability to regain control of the sphincters or respiratory muscles and restore hand function represent very important achievements.

No previous study has analyzed the functional and histological effects of estrogen in SCI in a single experiment in the same sample of rats. The objective of the present study was to evaluate the functional and histological results of six weeks of estrogen treatment immediately after a standard experimentally induced spinal cord lesion in rats. Our hypothesis was that estrogen would exhibit a neuroprotective effect that could be demonstrated by histological outcomes and functional evaluations.

## METHODS

### Ethics, study design, animals and allocation

This was an experimental, controlled study with Wistar rats that was performed in the laboratory of the Hospital das Clínicas da Faculdade de Medicina da Universidade de São Paulo Instituto de Ortopedia e Traumatologia (IOT-HCFMUSP) (São Paulo, Brazil). The study protocol was approved by the Institutional Review Board and we certify that all applicable institutional and governmental regulations concerning the ethical use of animals in experiments were followed during the course of this research.

Twenty male Wistar rats were divided in two groups, both of which were subjected to standard experimentally induced spinal cord lesions with the NYU Impactor device as described below. Ten animals received estrogen therapy (with intraperitoneal 17-beta estradiol) under sedation and the 10 rats in the control group were not treated. The sample size was based on previous studies 4,5.

The allocation of the animals to each group was concealed from the surgeon performing the experimental SCI (OL). The researcher administrating the estrogen therapy was not blind to the allocation because the animals in the control group did not receive injections. However, the allocation was concealed from the researchers who were involved in both the histological and functional evaluations.

The 20- to 21-week-old rats, weighing 300 g to 420 g, were all healthy, had a normal gait and were obtained from the university vivarium. Five animals from the same litter were housed in each cage in the laboratory, with adequate feeding and hydration. The animals were manipulated and stimulated to move before the experiment so that they could adapt to contact with the researchers and to the motor function evaluation after SCI. All experiments were performed at the same time of the day in both groups to avoid interfering with the day-night cycle. All rats were euthanized on the 42^nd^ day after the experiment.

Death after SCI, autophagic or mutilating behavior and macroscopic spinal anomalies were the exclusion criteria for our study. If the rats exhibited normal movement after the experimental lesion (21 points on the Basso Beattie and Bresnahan (BBB) scale) or there was a problem with the NYU impactor, the animals were excluded from the analysis. 

### Anesthesia, laminectomy and SCI

Intraperitoneal injections of xylazine (10 mg/kg) and ketamine (50 mg/kg), followed by subcutaneous injections of lidocaine hydrochloride with epinephrine, were used for anesthesia before the experiment. A veterinarian monitored all of the procedures to ensure the absence of reflexes. Surgery was performed using aseptic techniques.

A previously described protocol for weight-drop SCI 4,5 was used to produce a moderate lesion. The lesion was generated with the NYU Impactor (New York University Spinal Cord Contusion System) and a 10-g impact rod from a standardized height of 12.5 mm, which compressed the spinal cord for 15 seconds. The lesion was produced at T10.

### The experimental intervention: estrogen injection

Estrogen was administered intraperitoneally as 17-beta estradiol (Drogavet, Curitiba), only to animals in the study group. A dose of 4 mg/kg was given immediately following SCI, after skin sutures were applied 7-9, while the animal was still under anesthesia and sedated. Estrogen was administered by the veterinarian who was caring for the animals.

### Animal care after SCI

After cleaning the surgical scar, a layer of topical ointment (fibrinolysin, deoxyribonuclease and chloramphenicol) was applied. The animals then immediately received cefazolin sodium (Cezolin, BioChimico, Rio de Janeiro) intraperitoneally (5 mg/kg) as a prophylactic. Animals presenting an infection (inflammatory signs such as purulent secretions or abnormal urine) received an antibiotic for 10 days and were excluded from the statistical analysis.

For pain relief, the rats in both groups were intramuscularly administered a non-steroidal anti-inflammatory agent (2 mg/kg of meloxicam, once daily, for 7 days), along with tramadol hydrochloride (5 mg/100 g, once daily for 5 days). The injections were administered in the deep muscles of the lower limbs (thighs).

The animals' bladders were manually emptied at least twice daily until the animals regained bladder function. The rats were housed in their original cages (40×60 cm for a group of five), with food and water available ad libitum (offered as soon as the animal was fully awake).

### Functional and motor-evoked potential evaluations

Function was evaluated using the BBB scale 4,5 by two trained evaluators who were blind to the animal allocation. In the case of a disagreement, the lowest score between the two was registered for statistical analysis. The BBB evaluation took place on the 2^nd^, 7^th^, 14^th^, 21^st^, 28^th^, 35^th^ and 42^nd^ days after SCI. The rats were stimulated to move by gentle touches and the evaluations took four to five minutes per rat.

**Figure 1:**
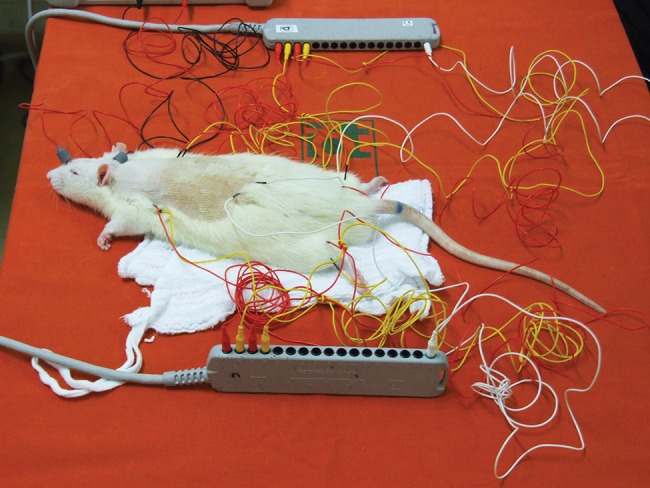
Anesthetized Wistar rat undergoing functional evaluation with the motor evoked potential test after experimental spinal cord lesion.

On the 42^nd^ day after SCI, the rats were anesthetized (with ketamine and xylazine, as described) and evaluated using the evoked potential test (MEP) 7 through transcranial electrical stimulation at the cortical level, with the responses captured at the muscular level (Figure 1). The electrodes were positioned in the semitendinosus and the biceps muscles of the thigh. The test monitored the response to the stimulus for 100 ms at most. For the average latency calculation, we used this maximum time of observation when no response was observed and the amplitude was recorded as zero. During observation of the response to electrical stimulation, the latency and amplitude were recorded at the time the signal was observed. The amplitude was registered in millivolts and the latency was recorded in milliseconds.

The MEP and BBB tests were performed by a physician blinded to the animal allocation, according to methods described by others 6,14,15.

### Euthanasia

All rats were sedated and anesthetized prior to euthanasia, which took place on the 42^nd^ day after the experiment. Euthanasia consisted of transcardial perfusion with a 4% paraformaldehyde solution and intravenous administration of thiopental (65 mg/100 g) and potassium chloride.

### Histological analysis

After euthanasia, the vertebral spine was exposed through an extensive dorsal incision and a 2.5-cm segment of the spinal cord from T8 to T12 was removed with scissors, including the focus of the lesion. Visual macroscopic evaluation of the spinal cord at the contusion site was performed to check for any anomalies (exclusion criteria) 5. The extracted segment was fixed and prepared for histological analysis, as described by Cristante et al. 4.

Thoracotomy and laparotomy enabled the inspection of the lungs, abdomen and bladder and the researcher was looking for signs of empyema, condensation, a flaccid neurogenic bladder (with increase in volume) or hyperemia and hematuria 5.

The fragments were fixed in formalin (10%), identified, dehydrated in alcohol baths, diaphanized in xylol and impregnated with paraffin. Five-micron-thick sections were cut (1-cm caudally and 1-cm cranially from the center of the lesion) for histological analysis and stained with hematoxylin-eosin (HE) 4,5.

An experienced pathologist (not an author) performed the microscopic evaluations and evaluated the following parameters: necrosis, hemorrhage, hyperemia, cellular infiltration and axonal degeneration. These variables were scored as zero (absence), 1 (discrete), 2 (moderate) and 3 (severe).

**Figure 2:**
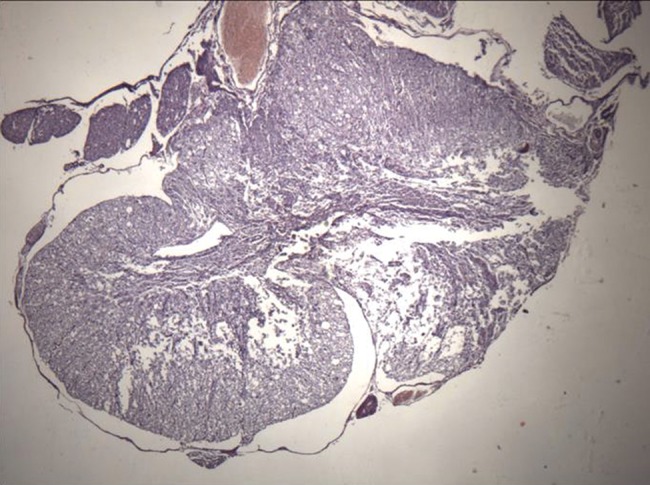
Moderate cellular infiltration and cystic degeneration of the spinal cord tissue in a Wistar rat submitted to experimental spinal cord injury (40 x magnification, hematoxylin-eosin staining). The image refers to the animal number 7 from the estrogen group.

As described previously 4,5, the proximal and distal portions of the spinal cord were cut and fixed in osmium tetroxide solution (2%) and then stained with toluidine blue (at 1%, Figure 2). Two fields were selected from transverse sections of the spinal cords; the sections were 2 microns thick and were located 1 mm distal and 1 mm proximal from the center of the lesion. The regenerated axon fibers were counted in the photos using the Sigma Scan Pro 5.0 software; only neurons with a diameter greater than or equal to 15 µm were considered during counting. The following formula was used: IR = (number of axons in the distal area/number of axons in the proximal area) × 100.

### Statistical analysis

The primary outcome considered in this study was the BBB score on the 42^nd^ day. The secondary outcomes were the MEP and results of histological analysis (considered as a subjective measure in the HE staining analysis and an objective measure in the toluidine blue staining).

The continuous data were tested for normality using the Kolmogorov-Smirnov test. If the distribution was normal, Student's t test was used for comparisons between groups. The descriptive statistics were presented as the means and standard deviations (SD). For the evaluation over time, an analysis of variance (ANOVA) with repeated measures was used. The ordinal data in the histological evaluation were analyzed with the Mann-Whitney test. A Chi-squared test was used to analyze the categorical data.

The Statistical Package for Social Sciences (SPSS) software, version 19.0 for Windows, was used in the statistical analysis. *P*-values that were less than or equal to 0.05 were considered significant.

## RESULTS

There was no death in any of the groups before the 42^nd^ day. It was possible to obtain complete spinal cord lesions in all rats. Autopsy revealed no empyema or condensation in the abdomens and lungs and no cases of neurogenic bladder, hyperemia or hematuria were detected. The SCI was effective in all animals and all rats were included in the analyses.

After SCI, the week-by-week BBB scores showed evident improvements in the functional evaluation of all rats from the fourth week through the end of the study (*p*=0.038), although significantly better scores were observed in the experimental group of rats, which received the estrogen treatment (Table 1).

The MEP evaluation also showed an enormous difference between the treated and untreated animals. After SCI, the estrogen-treated rats performed significantly better than the control rats, considering both the latency (time taken from the impulse to be transmitted from the head to the limbs; *p*=0.000) and amplitude values (a reflection of the number of axonal fibers involved in the impulse transmission; *p*=0.007) (Table 2). The exam showed that in the estrogen-treated rats, the electric impulse travelled more than 17 times faster between the limbs and the head, with a 7-fold increase in amplitude. In the control group, 50% of the rats had no response of any type in the MEP. However, there was a bit of noise in both the average latency and amplitude values. We performed a comparative analysis of the latency between groups, substituting the value of 100 ms with 500 ms for the 5 rats in the control group (time observed in the latency analysis) and the average value was reduced. However, the estradiol-treated group still showed the lowest latency, with a statistically significant difference (*p*<0.05) compared to the control group (data not shown).

**Figure 3:**
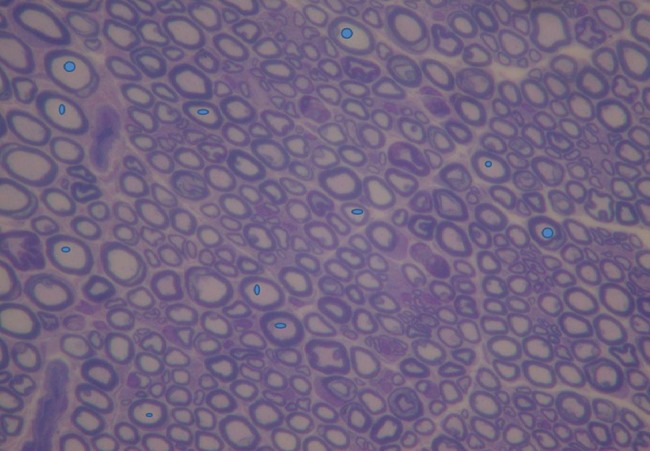
Photomicrograph showing spinal cord tissue stained with toluidine blue in a Wistar rat submitted to experimental spinal cord injury (100 x magnification). Viable axon neurons are stained blue for evaluation.

There was no significant difference between groups in the histological tissue analysis for the following variables: necrosis, hemorrhage, hyperemia, axon degeneration (by HE) and cellular infiltration (Table 3). Figure 3 shows examples of the tissue abnormalities after SCI. In the axon analysis with toluidine blue staining, the control group had a significantly lower neuron count than the treated group. The diameter of the axons was also significantly smaller in the control group compared to the estrogen-treated group (Table 4).

## DISCUSSION

The effects of estrogen in nerve regeneration or protection have been studied throughout the last decade using various experimental models 7-10,13, and the results have shown the benefits of this hormone in different outcomes. In brain cells, estrogen protects against glutamate-induced cell death and oxidative stress. Estrogen also improves recovery after traumatic brain injury, cerebral ischemia and peripheral nervous system lesions 12. The neuroprotective effects of estrogen are evident both before and after the neurological lesion, as well as in advanced phases of neurological tissue damage 8,16.

This study is the first to analyze functional recovery using two instruments (the BBB evaluation and the MEP exam) and two different histological evaluations. The follow-up period was also extended to six weeks, similar to the study by Hubscher et al. 16 In their study, the functional results in the treated group persisted throughout the follow-up period. Similar studies 7-10,13, have also demonstrated benefits from the same dosage of estrogen (4 mg/kg) in rats with SCI 7-9. The findings in this study are consistent with the most recent scientific literature 7-10,12,13, and our study is original because it combines histological and functional analysis.

This study showed significantly higher BBB scores beginning at the fourth week following SCI, which is different from the findings reported by Yune et al. 10 and Ritz and Hausmann 8, where the animals improved earlier. The latter study actually reported lower scores after the fourth week. In addition, the animals in the study by Sribnick et al., 2005 7, were sacrificed at 48 hours after SCI, whereas in this present study, the animals were sacrificed only at the end of the sixth week and they still maintained the benefits of estradiol administration. Longer-lasting benefits of estrogen therapy were also reported in the study by Hubscher et al., 2010 16, which also extended the experiment until the sixth week after injury. Olsen et al. 13 administered estrogen for 21 days after SCI and obtained good cellular physiology results.

A single dose of estrogen was used in this experiment and the benefits were only observed beginning at the fourth week. Other authors 8,10,13 have administered estrogen for longer periods and obtained good cellular physiology results. One possible explanation for the excellent results of estrogen administration in our study is that a higher dose was used soon after the lesion was induced 11. Therefore, although this study provides new evidence for the benefits of estrogen in SCI in three different outcomes, one limitation of our experiment is that the early *versus* late administration of the drug was not tested. Instead of using a single dose of estrogen, a prolonged treatment period might have produced even stronger results. According to Sribnick et al. 9, even chronic cases of SCI seem to show motor function improvement after estrogen therapy. However, the early *versus* late benefits of estrogen administration could not be evaluated due to the study design.

The current study highlights the pioneer use of the MEP test in experiments with estrogen; this approach represents a more objective evaluation of functional results than does the BBB test because the BBB test is observer-dependent. The speed at which the electric signal from the neuron travels, expressed as latency and the ability of the fibers to recover, expressed by the amplitude, are clinically correlated with clinical exams and the MEP is not limited in cases of anterior spinal cord syndromes, as is the somato-sensitive exam 6. Therefore, the MEP test is a promising tool in future studies on the effect of estrogen in SCI recovery.

Specifically, in this study, the MEP results obtained in the control group (higher latency and lower amplitude values) may serve as a direct and objective indicator of the trauma to the spinal cord. The higher latency in the control group compared with the estrogen group could be due to a loss of myelin, which, in turn, is a consequence of the neural tissue damage and compromised microcirculation. Moroever, the lower amplitude values in the control group compared to those in the treated group may be the ultimate consequence of axonal degeneration after the primary and secondary changes in the neural tissue (including neuronal death and apoptosis), leading to a reduction in the number of functioning neurological paths (axons).

Another important feature of our study is that in addition to the histological analysis of necrosis, hemorrhage, hyperemia and cellular infiltration with HE staining, which confirmed the generation of SCI, toluidine blue was used to count the axons and neurons in a much more objective evaluation 20. The HE staining did not show significant differences in necrosis, hemorrhage, hyperemia, axon degeneration or cellular infiltration between the treated rats and the controls, which is likely because the HE evaluation was not sensitive enough to detect the effects of a moderate lesion caused by the NYU Impactor 5. One could criticize the scoring system used in this study for the histological evaluation of necrosis, hemorrhage, hyperemia and cellular infiltration; indeed, this analysis is subject to some bias in the personal evaluation of the slides, even when using a scoring system. However, no other objective evaluation of the presence of these histological variables is currently available. Nevertheless, the axon and neuron counts, which represent a much more objective evaluation, showed significant differences that might explain the functional recovery observed in the rats receiving estrogen.

Science is unraveling promising frontiers in the treatment of SCI, a field with limited resources beyond palliative care. Animal models are important tools in the search for effective treatments. Future studies using 17-beta estradiol, a widely available endogenous hormone and sensitive and objective evaluation tests could possibly provide results that would allow the initiation of tests with humans. Moreover, it is necessary to examine different dosages and times of administration, similar to the doses that have already been proven safe in clinical use (such as those typically used with estrogen replacement therapy) to accelerate the translation of 17-beta estradiol as a therapeutic intervention for use in SCI patients. Additionally, the investigation of non-feminizing congeners of 17-beta estradiol, such as 17-alpha estradiol, could be useful.

The administration of estrogen immediately after SCI showed neuroprotective effects, as demonstrated by functional motor recovery of the treated animals, beginning at the fourth week after lesion formation. The hypothesis of a neuroprotective effect of estrogen was confirmed by the functional evaluations, but not by the tissue histological evaluations.

## Figures and Tables

**Table 1:** Mean BBB scores in the treated and control groups for each week (n=10 for each group).

	Group	Mean	Standard deviation	*p*
Week 1	Estradiol	0.6	0.699	0.791
		Control	0.7		0.949
Week 2	Estradiol	1.8	1.317	0.159
		Control	2.9		1.969
Week 3	Estradiol	5.2	1.989	0.082
		Control	3.7		1.636
Week 4	Estradiol	8.1	2.726	0.038
		Control	5.7		2.003
Week 5	Estradiol	11.4	2.951	0.014
		Control	8.5		1.269
Week 6	Estradiol	15.1	2.132	0.000
		Control	9.3		1.494

**Table 2:** Amplitude and latency values obtained in the evoked potential test in the hind limbs of the rats in each group.

	Group	Mean	Standard deviation	*p*
Latency	Estradiol	3.68	0.80	0.000
		Control	53.06		48.16
Amplitude	Estradiol	14.34	16.90	0.007
		Control	2.91		0.95

**Table 3:** Means and standard deviation of the histological scores in each group.

	Group	Mean	Standard deviation	*p*
Necrosis	Estradiol	1.90	0.568	0.125
		Control	1.50		0.527
Hemorrhage	Estradiol	1.90	0.568	0.957
		Control	1.90		0.316
Hyperemia	Estradiol	2.30	0.823	0.188
		Control	1.90		0.568
Axon degeneration	Estradiol	1.60	0.843	0.619
		Control	1.70		0.675
Cellular infiltration	Estradiol	1.70	0.949	0.813
		Control	1.80		1.033

**Table 4:** Mean and standard deviation of the number of distal/proximal fiber counts in each group.

	Group	Mean	Standard deviation	*p*
Number of fibers	Estradiol	92.632	16.164	0.00
		Control	56.933		13.032
Diameter of fibers	Estradiol	92.427	19.097	0.00
		Control	55.132		20.075
